# Tin phosphide-based anodes for sodium-ion batteries: synthesis *via* solvothermal transformation of Sn metal and phase-dependent Na storage performance

**DOI:** 10.1038/srep26195

**Published:** 2016-05-18

**Authors:** Hyun-Seop Shin, Kyu-Nam Jung, Yong Nam Jo, Min-Sik Park, Hansung Kim, Jong-Won Lee

**Affiliations:** 1 Energy Efficiency Research Division, Korea Institute of Energy Research, 152 Gajeong-ro, Yuseong-gu, Daejeon 34129, Republic of Korea; 2 Department of Chemical and Biomolecular Engineering, Yonsei University, 50 Yonsei-ro, Seodaemun-gu, Seoul 03722, Republic of Korea; 3 Advanced Batteries Research Center, Korea Electronics Technology Institute, 25 Saenari-ro, Bundang-gu, Seongnam 13509, Republic of Korea; 4 Department of Advanced Materials Engineering for Information and Electronics, Kyung Hee University, 1732 Deogyeong-daero, Giheung-gu, Yongin 17104, Republic of Korea; 5 New and Renewable Energy Research Division, Korea Institute of Energy Research, 152 Gajeong-ro, Yuseong-gu, Daejeon 34129, Republic of Korea; 6 Department of Advanced Energy and Technology, Korea University of Science and Technology, 217 Gajeong-ro, Yuseong-gu, Daejeon 34113, Republic of Korea

## Abstract

There is a great deal of current interest in the development of rechargeable sodium (Na)-ion batteries (SIBs) for low-cost, large-scale stationary energy storage systems. For the commercial success of this technology, significant progress should be made in developing robust anode (negative electrode) materials with high capacity and long cycle life. Sn-P compounds are considered promising anode materials that have considerable potential to meet the required performance of SIBs, and they have been typically prepared by high-energy mechanical milling. Here, we report Sn-P-based anodes synthesised through solvothermal transformation of Sn metal and their electrochemical Na storage properties. The temperature and time period used for solvothermal treatment play a crucial role in determining the phase, microstructure, and composition of the Sn-P compound and thus its electrochemical performance. The Sn-P compound prepared under an optimised solvothermal condition shows excellent electrochemical performance as an SIB anode, as evidenced by a high reversible capacity of ~560 mAh g^−1^ at a current density of 100 mA g^−1^ and cycling stability for 100 cycles. The solvothermal route provides an effective approach to synthesising Sn-P anodes with controlled phases and compositions, thus tailoring their Na storage behaviour.

In recent years, strong demand for affordable energy storage technologies has driven extensive research on rechargeable sodium (Na)-ion batteries (SIBs) operating at ambient temperature^1^,^2^,^3^,^4^,^5^. SIBs are believed to offer great benefit in material cost over conventional lithium (Li)-ion batteries (LIBs) due to the natural abundance of Na. Therefore, they have been considered promising battery systems for large-scale stationary energy storage applications combined with renewable energy power plants in which the major concern is a battery’s cost rather than its specific energy density^1^,^2^,^3^,^4^,^5^. The operation mechanism of SIBs is similar to that of LIBs; however, finding electrode materials that are capable of reversibly accommodating a large amount of Na has proved to be quite challenging. In particular, there is a limited choice of candidate materials that can be used as an anode (negative electrode) for SIBs^6^,^7^. Carbonaceous materials such as hard carbon and amorphous carbon deliver reversible capacities as high as 150–350 mAh g^−1^, depending on their crystallinity and microstructures^8^,^9^,^10^,^11^. Turning to metallic materials, theoretical and experimental studies have demonstrated that Sn and its alloys (Sn-Cu, Sn-Ni, *etc.*) have the ability to store Na *via* electrochemical alloying reactions, leading to much higher specific capacities (500–700 mAh g^−1^) in comparison to carbonaceous materials^12^,^13^,^14^,^15^,^16^,^17^,^18^,^19^. However, large volume changes of these materials (*e.g.*, ~420% for Sn) upon sodiation and desodiation result in significant capacity decay during repeated discharge–charge cycles, which hinders their practical use in SIBs^13^,^18^,^20^.

A series of interesting studies have been published very recently, reporting that tin phosphide (Sn_4_P_3_) prepared by high-energy mechanical milling can deliver a high capacity and show improved cyclability compared with pure Sn^21^,^22^,^23^. To explain the highly stable cycling performance of Sn_4_P_3_, Qian *et al.*^21^ suggested a synergistic role of Sn and P components as follows. Upon the sodiation of Sn_4_P_3_, Sn transforms to Na_15_Sn_4_ nanoparticles that can provide a conductive network to facilitate the formation of Na_3_P. At the same time, the finely dispersed Na_3_P phases would prevent the aggregation of the Na_15_Sn_4_ and/or Sn nanoparticles and help maintain their homogeneous distribution in the electrode. Furthermore, Sn_4_P_3_ offers the important advantage of operating at relatively low potentials of <0.5 V *vs.* Na/Na^+^, thus enabling the design and construction of SIBs with high energy density.

Herein, we report Sn-P compounds synthesised *via* a facile solvothermal route and their electrochemical behaviour as SIB anodes. Sn-P compounds were prepared by the solvothermal reaction of Sn metal and red P in ethylenediamine under various conditions and their Na storage behaviour was investigated to determine the synthesis–phase (structure/composition)–property relationship. As will be shown later, the Na storage capability of the solvothermally synthesised Sn-P compound strongly depends on its phase and composition. The Sn-P compound prepared under an optimised solvothermal condition shows a high reversible capacity of ~560 mAh g^−1^ and excellent cycling stability for 100 cycles as a result of its controlled phase and composition.

## Results

Traditional methods of synthesising Sn-P compounds are based on high-energy mechanical milling of Sn and P components^24^,^25^,^26^. In this work, Sn-P compounds were synthesised *via* a solvothermal method, which is known to allow for easy and precise control of the crystallinity, phase, and composition of reaction products^27^,^28^. Sn-P compounds were obtained through the solvothermal reaction of metallic Sn and red P powders in ethylenediamine at elevated temperatures^27^. To study the synthesis–phase (structure/composition)–property relationship of Sn-P compounds, the solvothermal synthesis was performed using various combinations of reaction temperature and time. For simplicity, the solvothermally synthesised Sn-P powders will hereafter be referred to as SP(*T*/*t*), where SP stands for tin phosphide, and *T* and *t* represent the solvothermal reaction temperature (°C) and time (h), respectively. For example, SP(160/20) represents a tin phosphide powder prepared through solvothermal treatment at 160 °C for 20 h.

**Figure 1:**
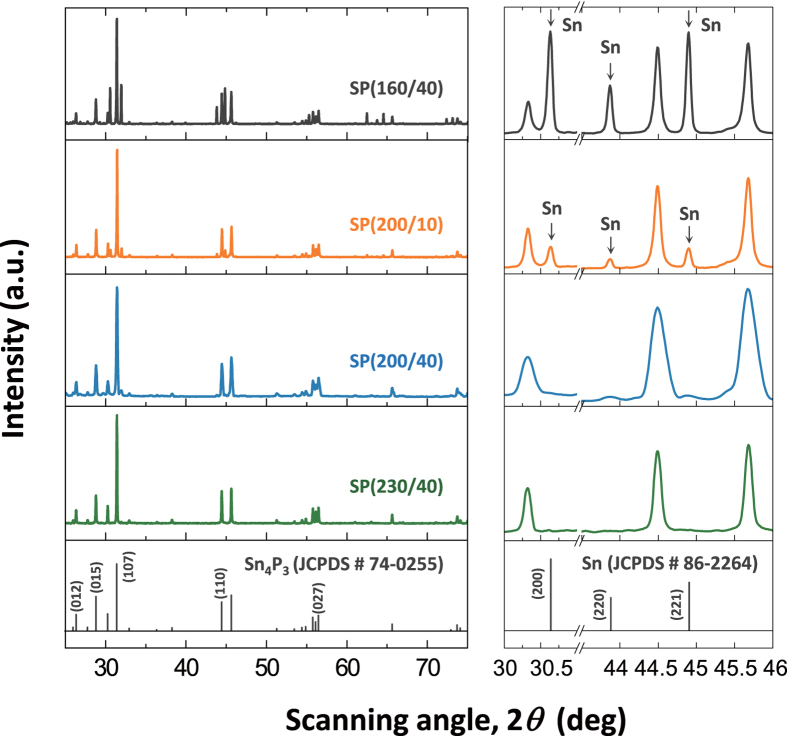
Structural characterisation of the solvothermally synthesised Sn-P compounds. XRD patterns of SP(160/40), SP(200/10), SP(200/40), and SP(230/40). The reference XRD patterns for Sn_4_P_3_ (JCPDS No. 74-0255) and Sn (JCPDS No. 86-2264) phases are presented.

The crystal structure and phase of the Sn-P powders synthesised with various solvothermal parameters were characterised by X-ray diffraction (XRD) analysis, and the results are presented in Fig. 1. The XRD patterns of SP(200/40) and SP(230/40) exhibit the diffraction peaks corresponding to a rhombohedral Sn_4_P_3_ phase (space group *R-3‒m*, JCPDS No. 74-0255). On the other hand, SP(160/40) and SP(200/10) are found to contain a considerable amount of metallic Sn species (space group *I4*_*1*_/amd, JCPDS No. 86-2264). The XRD analysis demonstrates that the intermetallic Sn_4_P_3_ phase with a layered structure can be successfully produced *via* a simple solvothermal route and that the solvothermal parameters, such as temperature and time, play an important role in determining the phase and composition of the Sn-P compound.

It has been known that, when a mixture of Sn and red P powders in ethylenediamine is subjected to solvothermal treatment at elevated temperatures, red P becomes soluble in ethylenediamine, and then, the dissolved P species reacts with solid Sn and diffuses towards the interior of the Sn particles to form Sn-P alloys^27^. The XRD results of Fig. 1 indicate that the formation of Sn_4_P_3_-based Sn-P compounds can be achieved upon solvothermal treatment at *T* ≥ 200 °C for *t* ≥ 40 h. As seen in Fig. 1, when both the above critical temperature and time were not reached, a large amount of unreacted Sn species was still present in the solvothermally treated samples, *i.e.*, SP(160/40) and SP(200/10). The intensities of the diffraction peaks for Sn are higher in the pattern of SP(160/40) than in that of SP(200/10), suggesting a larger amount of residual Sn species in SP(160/40). The weight fractions of the crystalline Sn_4_P_3_ and Sn phases in the Sn-P compounds synthesised under various conditions were quantitatively determined using the reference intensity ratio (RIR) procedure. Table 1 confirms that the Sn_4_P_3_ phase fraction rises with increasing temperature and time and that the Sn-P compounds solvothermally synthesised at *T* ≥ 200 °C for *t* = 40 h are mostly composed of Sn_4_P_3_, *i.e.*, 98.3% and 98.5% Sn_4_P_3_ in SP(200/40) and SP(230/40), respectively.

**Figure 2:**
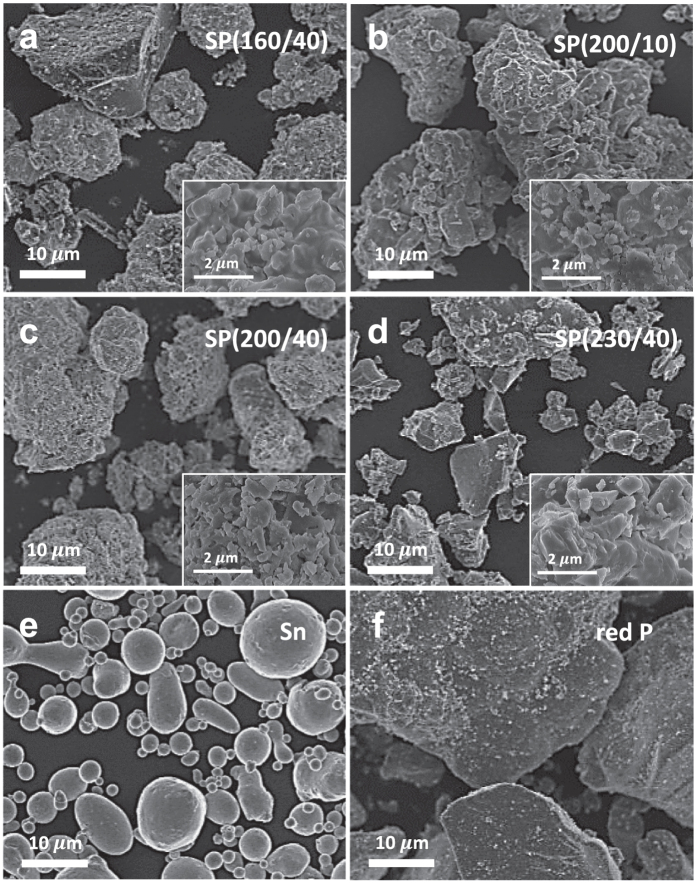
Morphological analysis of the solvothermally synthesised Sn-P compounds. SEM micrographs of (**a**) SP(160/40), (**b**) SP(200/10), (**c**) SP(200/40), and (**d**) SP(230/40). For comparison, the SEM images of Sn and P used for solvothermal synthesis are presented in (**e**,**f**), respectively. High-magnification SEM images are shown in the respective insets.

**Figure 3:**
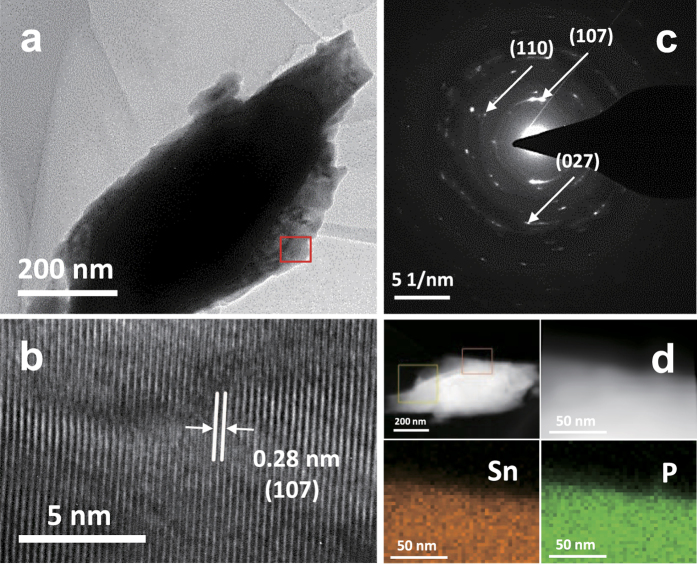
TEM analysis of the solvothermally synthesised Sn-P compound. (**a**) Low-magnification TEM image of SP(200/40) and (**b**) high-resolution TEM image of the region marked by square in (**a**). (**c**,**d**) Present the SAED pattern and the elemental distributions of Sn and P, respectively.

Figure 2a–d show SEM images of the solvothermally synthesised Sn-P powders. For comparison, the SEM micrographs of Sn and red P powders used as the precursors in the solvothermal process are also given in Fig. 2e,f, respectively. All of the Sn-P powder samples have irregular surface morphologies and consist of small particles with sizes of several hundred nanometers. It is interesting to note that the particle shapes of the Sn-P compounds appear quite different from those of metallic Sn (Fig. 2e) and red P (Fig. 2f), which reflects that the solvothermal transformation of Sn metal to Sn_4_P_3_ is accompanied by significant structural and morphological changes. For comparison, the SEM micrograph of SP(160/10) is presented in Supplementary Fig. S1, and the particles are similar in appearance to the Sn precursors. More detailed morphological and microstructural information of the solvothermally synthesised Sn-P powder was obtained by transmission electron microscopy (TEM) (Fig. 3). The high-resolution TEM image of SP(200/40) (Fig. 3b) clearly shows lattice fringes with a *d*-spacing value of 0.280 nm corresponding to the (107) plane of the rhombohedral Sn_4_P_3_ phase. The characteristic diffraction rings for the (107), (110), and (027) planes are also shown in the selected area electron diffraction (SAED) pattern of Fig. 3c, which is in accordance with the XRD results (Fig. 1). Energy dispersive X-ray spectroscopy (EDS) mapping analysis of Fig. 3d confirms that Sn and P elements are uniformly distributed throughout the SP(200/40) particle.

**Figure 4:**
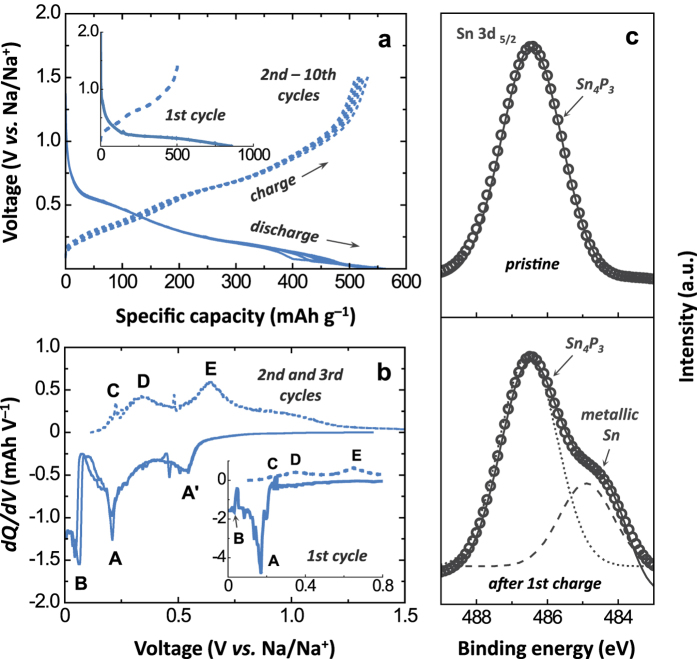
Electrochemical performance of Na-half cells assembled using the solvothermally synthesised Sn-P compound. (**a**) Galvanostatic discharge–charge curves of SP(200/40) measured at 100 mA g^−1^ and (**b**) differential capacity (d*Q*/d*V*) *vs.* voltage plots reproduced from the 1st, 2nd, and 3rd discharge–charge profiles in (**a**). (**c**) Presents Sn 3d XPS spectra for the pristine *a*nd recharged SP(200/40) anode.

To examine the Na storage capability of the solvothermally prepared Sn-P powders, we constructed and tested a Na half-cell using a metallic Na counter electrode. The electrolyte was 1 M NaClO_4_ in a mixed ethylene carbonate (EC)/propylene carbonate (PC) solvent (1:1 in volume) with 5 wt.% fluoroethylene carbonate (FEC). Here, FEC was used as an additive to promote the formation of stable solid–electrolyte interphase (SEI) layers on the surface of the Sn-P electrode as has been reported in earlier studies^22^,^23^,^29^,^30^. Prior to a comparative electrochemical study of the Sn-P compounds, we examined the Na storage behaviour of SP(200/40) comprising Sn_4_P_3_ (mixed with less than 2 wt.% Sn). Figure 4a illustrates the galvanostatic discharge (sodiation) and charge (desodiation) profiles of SP(200/40) measured for the first ten cycles. The experiments were conducted in a voltage range of 0.001–1.5 V *vs.* Na/Na^+^ at a current density of 100 mA g^−1^. The initial discharge capacity of SP(200/40) was determined to be 858 mAh g^−1^, which is comparable to the values reported in the literature, but is still lower than the theoretical value (1,080 mAh g^−1^) calculated assuming complete sodiation (Sn_4_P_3_ + 24Na^+^ + 24e^−^ → Na_15_Sn_4_ + 3Na_3_P). The initial charge capacity was measured to be 510 mAh g^−1^, resulting in a Coulombic efficiency of ~59%. The relatively low discharge capacity and Coulombic efficiency during the first cycle can be mainly attributed to the formation of NaF-like SEI layers upon the initial sodiation reaction^22^,^23^,^29^. To improve the initial Coulombic efficiency, therefore, comprehensive studies should be conducted to find optimum combinations of electrolyte salts, solvents, and functional additives that can lead to the formation of uniform, thin, and effective SEI layers on Sn-P anodes.

Plots of differential capacity (d*Q*/d*V*) *vs.* voltage (*V*) reproduced from the first discharge–charge curves are presented in Fig. 4b. On the first sodiation process, two reduction peaks are observed: (i) the sharp peak (A) at *~*0.18 V *vs.* Na/Na^+^ is associated with the sodiation reaction of pristine Sn_4_P_3_ (Sn_4_P_3_ → NaSn + Na_9_Sn_4_ + Na_3_P), and (ii) another small peak (B) at *~*0.04 V *vs.* Na/Na^+^ is linked to the further sodiation reaction of Na-Sn alloys (NaSn + Na_9_Sn_4_ + Na_3_P → Na_15_Sn_4_ + Na_3_P)^21^,^22^,^23^,^29^. On charging, the differential capacity plots are characterised by three peaks (C, D, and E). Peak C is due to the desodiation reaction of Na_15_Sn_4_ to Na-Sn alloys (NaSn and Na_9_Sn_4_), and peaks D and E correspond to the desodiation reactions of NaSn, Na_9_Sn_4_, and Na_3_P to form Sn_4_P_3_, Sn, and P^21^,^22^,^23^,^29^. To identify the phases present in the charged electrode, we performed *ex-situ* XRD analysis on the recharged SP(200/40) electrode. However, it was hard to observe clear diffraction peaks for Sn_4_P_3_, Sn and P phases in the XRD data of SP(200/40) recharged to 1.5 V *vs.* Na/Na^+^ (Supplementary Fig. S2), which indicates that the charged electrode is composed of amorphous-like and/or highly dispersed nanoparticles. The X-ray photoelectron spectroscopy (XPS) results of Fig. 4c provide clear experimental evidence that some of the Sn formed during the first discharge still remained in the electrode without converting back to Sn_4_P_3_ upon recharging. From the second sodiation process, the additional peak (A′) is observed at ~0.54 V *vs.* Na/Na^+^, which is attributed to the sodiation of Sn metal^29^. From the second cycle onwards, the SP(200/40) electrode was reversibly cycled, delivering discharge and charge capacities as high as 562 and 510 mAh g^−1^, respectively (Fig. 4a). The occurrence of three redox peak couples in the differential capacity plots of the subsequent cycles (Fig. 4b) indicate that the electrode is reversibly discharged and charged *via* the sodiation–desodiation reactions between (Na_15_Sn_4_ + Na_3_P) and (Sn_4_P_3_ + Sn + P).

**Figure 5:**
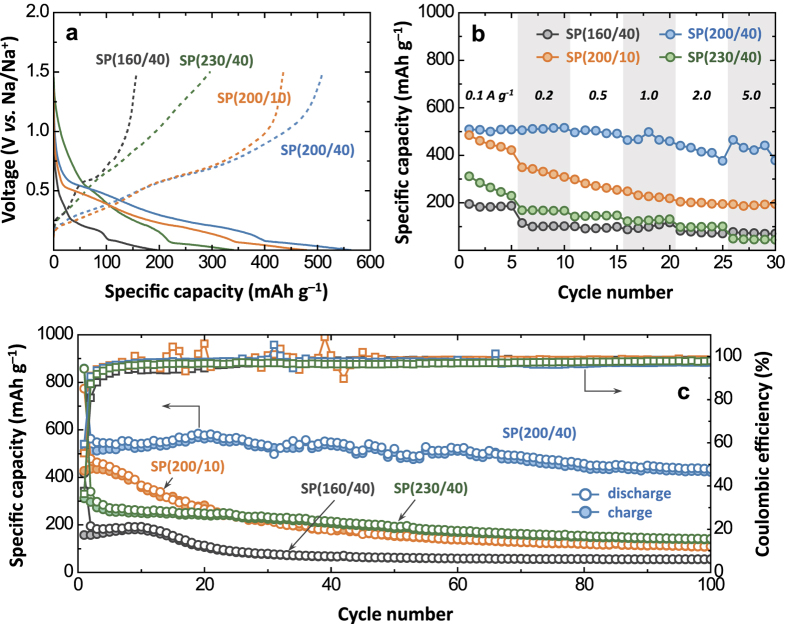
Comparative electrochemical study of the solvothermally synthesised Sn-P compounds. (**a**) Galvanostatic discharge–charge curves of SP(160/40), SP(200/10), SP(200/40), and SP(230/40) measured at 100 mA g^−1^. (**b**,**c**) Present the rate-capability and cycling performance, respectively.

The electrochemical properties of the Sn-P electrodes synthesised under various solvothermal conditions were characterised and compared in terms of capacity (Fig. 5a), rate-capability (Fig. 5b), and cyclability (Fig. 5c). Figure 5a illustrates the galvanostatic discharge–charge profiles of the Sn-P electrodes taken from the second cycle. The charge capacity determined at a current density of 100 mA g^−1^ increases in the order of SP(160/40) (157 mAh g^−1^) < SP(230/40) (311 mAh g^−1^) < SP(200/10) (427 mAh g^−1^) < SP(200/40) (510 mAh g^−1^). As demonstrated in Fig. 5b, the rate-capability of the Sn-P electrodes shows the same trend as that for the specific capacity. The SP(200/40) electrode exhibits higher capacity values than the others over the whole current range of 0.1–5.0 A g^−1^. At a high current density of 5.0 A g^−1^, in particular, the charge capacity of SP(200/40) was determined to be as high as 464 mAh g^−1^ (see the charge profiles in Supplementary Fig. S3). With the exception of SP(230/40), the capacity and rate-capability are correlated to the amount of Sn species left unreacted during solvothermal treatment; the smaller the amount of residual Sn, the better the Na storage performance is. Note that the discharge–charge behaviours of SP(160/40) containing ~58% Sn appear to be quite similar to those of Sn (Supplementary Fig. S4) rather than to those of Sn_4_P_3_. Both of the SP(160/40) and Sn electrodes exhibit discharge–charge profiles with two well-defined plateaus and delivered similar capacity values ranging between 150 and 250 mAh g^−1^.

The cycle performance results of the Sn-P electrodes are shown in Fig. 5c. The cells were discharged and charged with cut-off voltages of 0.001 and 1.5 V *vs.* Na/Na^+^ at a current density of 100 mA g^−1^. The SP(200/40) electrode exhibits excellent cyclability with capacity retention of 83% during 100 cycles, while the others show cycling stability inferior to that of SP(200/40), *i.e.*, 34% for SP(160/40), 25% for SP(200/10), and 44% for SP(230/40). In general, the large volume expansion of pure Sn upon sodiation (~420% for the transition of Sn to Na_15_Sn_4_) causes the pulverization-induced agglomeration of Sn particles, which in turn leads to considerable capacity decay during cycling^13^,^18^,^20^. In fact, the electrode made using the metallic Sn powder (used as the precursor for solvothermal synthesis) exhibited very poor cyclability (17% capacity retention after 50 cycles) as shown in Supplementary Fig. S4. Given that the particle dimension is a critical factor for electrochemical performance (most high-performance Sn electrodes have been reported for nanoscale materials), the microscale particle morphology of pure Sn (used in this work) seems to be responsible for its lower-than-expected capacity and cyclability.

Previous works^21^,^22^,^23^,^29^ have suggested that, for Sn_4_P_3_, amorphous P and Na_3_P phases surrounding Sn nanoparticles serve as a matrix to mechanically buffer the expansion of Sn, thereby mitigating the loss of structural integrity and electronic conducting paths of the electrode. In addition, the isolated Sn nanoparticles formed during the sodiation/desodiation process provide conductive channels to activate the electrochemical reaction of the P phase. Similar to the capacity and rate-capability results, the stable cyclability of SP(200/40) can be explained by the fact that the active material is mostly composed of Sn_4_P_3_ (98.3%) without a significant amount of residual Sn species. It is also speculated that the thick SEI layer on the electrode surface formed by the decomposition of FEC additive plays a beneficial role in mitigating the strain-induced degradation on cycling.

## Discussion

**Figure 6:**
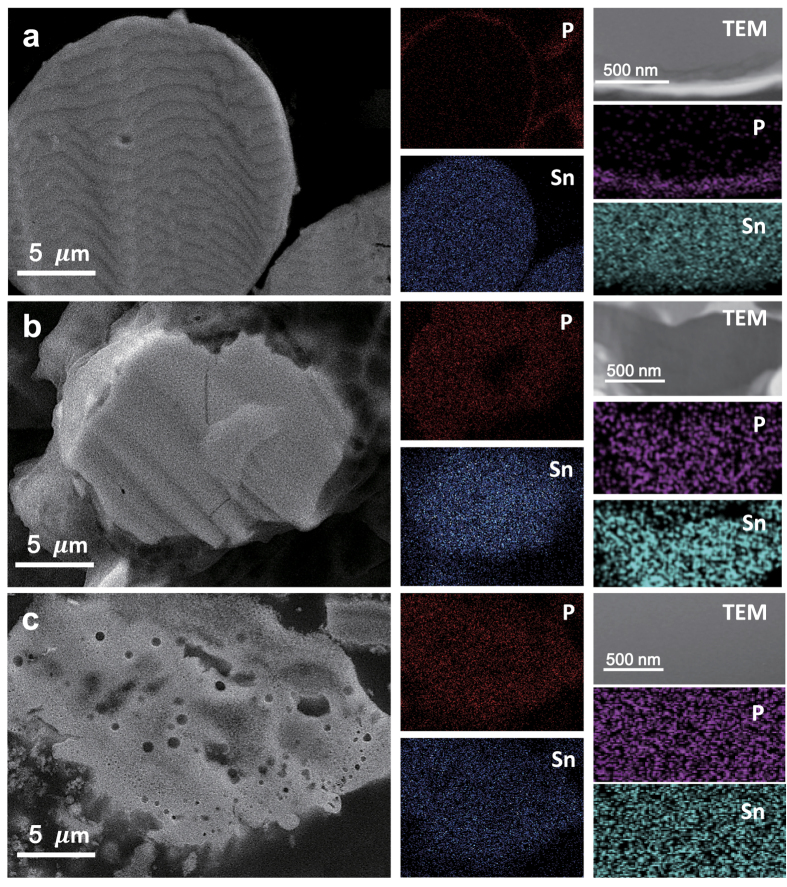
Elemental analysis of the solvothermally synthesised Sn-P compounds. Cross-sectional SEM micrographs of (**a**) SP(160/40), (**b**) SP(200/40), and (**c**) SP(230/40). The elemental distributions of Sn and P for the corresponding SEM and TEM images are presented.

The electrochemical test demonstrated that the solvothermally synthesised SP(200/40) compound (Sn_4_P_3_ mixed with ~1.7% Sn) would be a promising anode with high specific capacity, excellent rate-capability, and stable cyclability for SIBs. In Supplementary Table S1, the electrochemical Na storage performance of SP(200/40) developed in this work was compared with those of various phosphide-based materials reported in the literature. Turning back to Fig. 5, however, a question arises: why does SP(230/40) exhibit inferior Na storage performance to SP(200/40)? Note that there are no big differences in morphology and composition between SP(200/40) and SP(230/40) as shown in Fig. 2 and Table 1. To attempt to explain the observed performance difference of SP(200/40) and SP(230/40), more detailed microstructural and compositional analyses were conducted using SEM, TEM, and EDS. Figure 6 shows the cross-sectional SEM and TEM images of Sn-P particles with the EDS mapping results for Sn and P elements. In SP(160/40), P was mostly detected at the particle surface, indicating that the Sn-P compound consists of an outer shell of Sn_4_P_3_ (thickness ~300 nm) and a Sn core. This further confirms that the alloy formation reaction between Sn and P during solvothermal treatment proceeds in a radial direction from the surface of a particle towards its core. In both SP(200/40) and SP(230/40), on the other hand, Sn and P elements were found to be uniformly distributed throughout the particles, which means that any possible difference in particle morphology and/or elemental distribution could not give a reasonable answer to the above question.

Interestingly, we found a noticeable difference in relative concentrations of Sn and P from the EDS analysis conducted simultaneously with SEM/TEM measurements (Supplementary Fig. S5). The Sn/P ratio for SP(200/40) was determined to be ~1.25, which is similar to that of Sn_4_P_3_ (1.33), while the Sn/P ratio for SP(230/40) is ~0.6. This indicates that SP(230/40) contains a considerable amount of excess P species in addition to the crystalline Sn_4_P_3_ phase. Phosphorous species is much less conductive compared with Sn and Sn_4_P_3_ — the electrical conductivity of the solvothermally synthesised Sn-P compounds was measured at room temperature. The powder samples were uniaxially pressed into a disc under a pressure of 45 MPa, and then, their conductivity was measured by using a two-probe method. The results in Table 2 show that the conductivity of Sn-P compounds lies between those of Sn (462 S cm^−1^) and P (<1 mS cm^−1^ (out of range)). Note that SP(230/40) shows much lower conductivity (64 mS cm^−1^) than SP(200/40) (1.7 S cm^−1^), even though both of the compounds have almost the same amount of Sn_4_P_3_. As a result, we believe that excess P species is mainly responsible for the reduced electrical conductivity of SP(230/40) and thus for its inferior Na storage performance.

**Figure 7:**
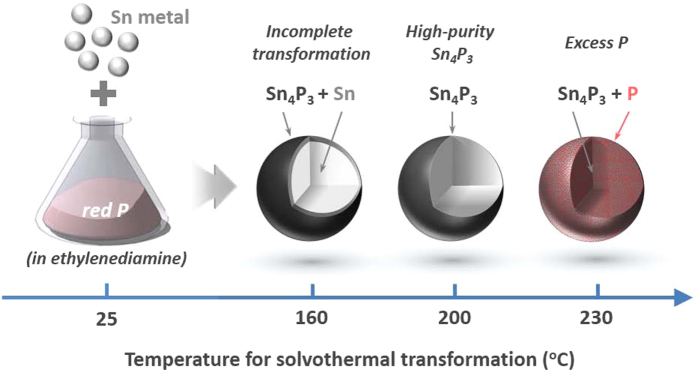
Effect of solvothermal temperature on the structure of the Sn-P compound. Schematic illustration showing the structures and phases of the Sn-P compounds synthesised through solvothermal treatment of Sn metal in the presence of P at various temperatures. The alloy formation reaction between Sn and P proceeds in a radial direction from the surface of a particle towards its core. If the solvothermal temperature is lower or higher than a critical value (200 °C), then the material contains unreacted Sn (in the core) or excess P species (throughout the particle), respectively.

In summary, we prepared Sn-P compounds for SIB anodes through a simple solvothermal transformation process of Sn metal in the presence of P. As schematically illustrated in Fig. 7, the temperature and time period used for solvothermal treatment were found to play a critical role in determining the phase and composition of the Sn-P compound and thus its electrochemical behaviour. In particular, when solvothermal synthesis was conducted at lower or higher temperatures than a certain critical value (*e.g.*, 200 °C), the resulting compound contains unreacted (residual) Sn or excess P species, respectively, which results in reduced Na storage performance. The battery tests indicate that the Sn-P compound prepared with the optimised solvothermal parameters (*T* = 200 °C and *t* = 40 h) shows promising electrochemical Na storage properties (high capacity as high as 562 mAh g^−1^ and excellent cycling stability for 100 cycles), proving its feasibility for use as SIB anodes. Nevertheless, the relatively poor cycling stability of Sn-P compound is still of concern. Therefore, rational approaches should be developed to further improve the cycle life of SIB anodes; for instance, designing hybrid electrodes in which an intercalation material and a conversion material can play a synergistic role may be an effective strategy to achieve both high capacity and good cycling performance^31^.

## Methods

### Materials preparation

Sn-P compounds were prepared *via* a solvothermal method using metallic tin (Sn) and red phosphorus (P) powders as precursors. In a typical procedure, 0.706 g (0.2 mol) of Sn and 0.207 g (0.3 mol) of P were mixed in 30 ml of ethylenediamine under magnetic stirring for 1 h. Then, the mixture was transferred into a Teflon-lined stainless-steel autoclave. The autoclave was sealed and maintained in the range of 160–230 °C for 10–40 h, and cooled naturally to room temperature. The product was collected by centrifugation and then washed with ethanol, HCl, and distilled water. Finally, the material was dried at 80 °C for 12 h under vacuum to evaporate the residual solvent.

### Materials characterisation

The phases and crystal structures of the synthesised powders were investigated by XRD (Rigaku 2500 D/MAX) with Cu-*K*_α_ radiation (*λ* = 0.15405 nm). The morphologies, microstructures, and compositions were analysed by SEM (Hitachi S-4800) and TEM (Tecnai TF30 ST) combined with EDS. XPS measurements were performed on a Sigma Probe with a monochromatic Al- *K*_α_ X-ray source. Electrical conductivity for powder samples was measured by using a two-probe method (Huvis ECT-200K). The powder samples were uniaxially pressed into a disc (7 mm in diameter and ~0.5 mm of thickness) under a pressure of 45 MPa.

### Electrochemical experiments

For the electrode preparation, a slurry was prepared by mixing the active material (60 wt.%), conductivity agent (20 wt.%, Super–P), and binder (20 wt.%, CMC and PAA) in distilled water. The slurry was then coated on a Cu foil, followed by drying under vacuum at 80 °C for 24 h. The electrodes were finally pressed with a twin roller. The electrochemical tests were performed using a coin-type cell (CR-2032) with a sodium metal counter electrode. The separator was a glass fibre sheet, and the electrolyte was 1 M NaClO_4_ dissolved in a mixed EC/PC solvent (1:1 in volume) with 5 wt.% FEC. The cell assembly was carried out in an argon-filled glove box. Galvanostatic discharge–charge measurements were conducted at room temperature using a battery tester (MACCOR-4000) with a voltage window of 0.001–1.5 V *vs.* Na /Na^+^ at a current density of 100 mA g^−1^.

## Additional Information

**How to cite this article**: Shin, H.-S. *et al.* Tin phosphide-based anodes for sodium-ion batteries: synthesis *via* solvothermal transformation of Sn metal and phase-dependent Na storage performance. *Sci. Rep.* **6**, 26195; doi: 10.1038/srep26195 (2016).

## Supplementary Material

Supplementary Information

## Figures and Tables

**Table 1:** Structural characterisation of the solvothermally synthesised Sn-P compounds.

Sample	Phase wt.%	
	Sn_4_P_3_	Sn
SP(160/10)	19.2	80.8
SP(160/20)	36.8	63.2
SP(160/40)	41.7	58.3
SP(200/10)	93.7	6.3
SP(200/20)	95.8	4.2
SP(200/40)	98.3	1.7
SP(230/10)	97.7	2.3
SP(230/40)	98.5	1.5

Weight fractions of the Sn_4_P_3_ and Sn phases in the Sn-P compounds synthesised with various solvothermal parameters. The phase wt.% values were determined by XRD analysis using the RIR procedure.

**Table 2:** Electrical property of the solvothermally synthesised Sn-P compounds.

Sample	Electrical conductivity (S cm^−1^)
Reference (Vulcan carbon)	13.8
Metallic Sn	462.0
SP(160/40)	68.9
SP(200/40)	1.7
SP(230/40)	6.4 × 10^−2^
Red P	<1 × 10^−3^ (out of range)

Electrical conductivity of SP(160/40), SP(200/40), and SP(230/40). The conductivity values of Vulcan carbon (reference), Sn, and P are presented.
