# Supplementary material for: Tin phosphide-based anodes for sodium-ion batteries: synthesis via solvothermal transformation of Sn metal and phase-dependent Na storage performance
Source: Sci Rep. 2016 May 18;6:26195. doi: 10.1038/srep26195 (PMC4870634; doi:10.1038/srep26195)
Supplement: Supplementary Information [file srep26195-s1.pdf]

SUPPLEMENTARY INFORMATION

Tin phosphide-based anodes for sodium-ion batteries: synthesis *via* solvothermal transformation of Sn metal and phase-dependent Na storage performance

Hyun-Seop Shin[1,2], Kyu-Nam Jung[1], Yong Nam Jo[3], Min-Sik Park[4], Hansung Kim[2*] & Jong-Won Lee[5,6*]

[1]Energy Efficiency and Materials Research Division, Korea Institute of Energy Research, 152 Gajeong-ro, Yuseong-gu, Daejeon 34129, Republic of Korea

[2]Department of Chemical and Biomolecular Engineering, Yonsei University, 50 Yonsei-ro, Seodaemun-gu, Seoul 03722, Republic of Korea

[3]Advanced Batteries Research Center, Korea Electronics Technology Institute, 25 Saenari-ro, Bundang-gu, Seongnam 13509, Republic of Korea

[4]Department of Advanced Materials Engineering for Information and Electronics, Kyung Hee University, 1732 Deogyeong-daero, Giheung-gu, Yongin 17104, Republic of Korea

[5]New and Renewable Energy Research Division, Korea Institute of Energy Research, 152 Gajeong-ro, Yuseong-gu, Daejeon 34129, Republic of Korea

[6]Department of Advanced Energy and Technology, Korea University of Science and Technology, 217 Gajeong-ro, Yuseong-gu, Daejeon 34113, Republic of Korea

Correspondence and requests for materials should be addressed to J.-W.L. (jjong277@kier.re.kr) or H.K. (elchem@yonsei.ac.kr)

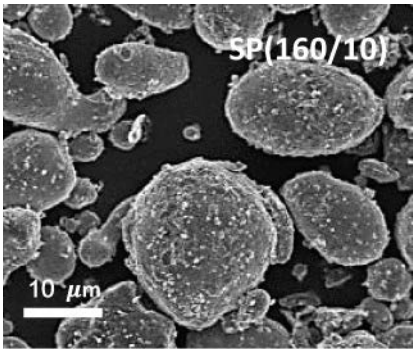

Supplementary Figure S1 | Morphological analysis of the solvothermally synthesised Sn-P compound. SEM micrograph of SP(160/10) synthesised through solvothermal treatment at 160 ºC for 10 h.

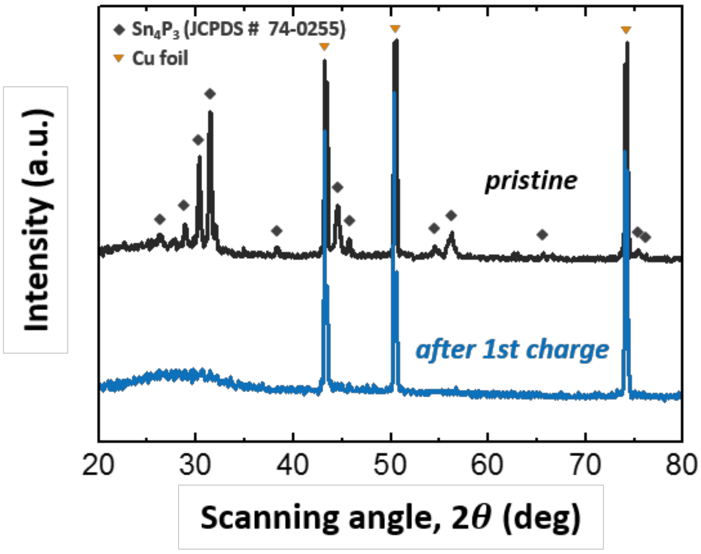

Supplementary Figure S2 | Structural characterisation of the solvothermally synthesised Sn-P compound. XRD patterns of the pristine and charged SP(200/40) electrodes.

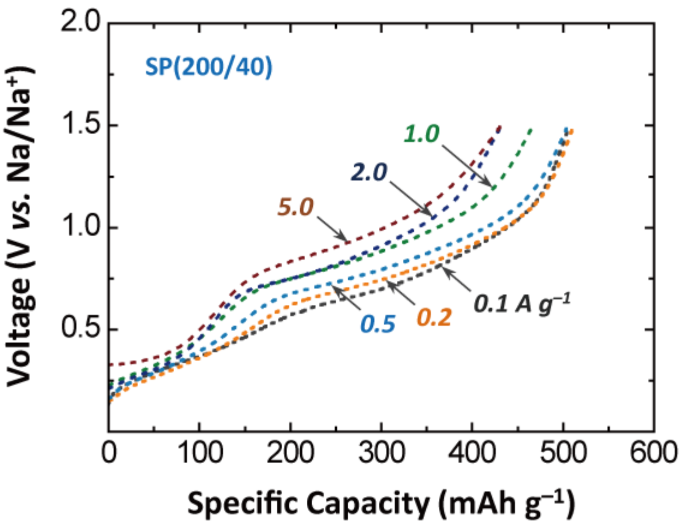

Supplementary Figure S3 | Electrochemical performance of a Na-half cell assembled using the solvothermally synthesised Sn-P compound. Galvanostatic charge curves of SP(200/40) measured at various current densities.

Overall, the charge profiles of SP(200/40) measured at various current densities are quite similar in shape, as shown in Fig. S3, which indicates the reliability of rate-performance measurements. However, they exhibit slightly different behaviours at the end of charge: for example, the charge curve at 5.0 A g^{-1} shows a voltage increase at a slower rate as compared with that measured at 2.0 A g^{-1}, resulting in almost the same capacity values for both current densities (when determined at a cut-off voltage of 1.5 V *vs.* Na/Na^{+}). A similar observation was also reported in the previous work[1] on the desodiation reaction of Sb nanorod array electrodes, but the origin for such anomaly has not been clearly understood yet.

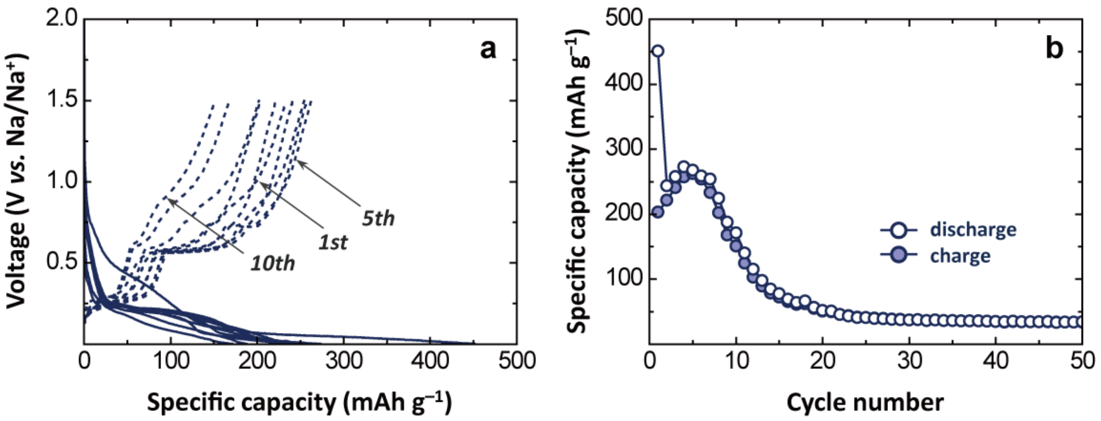

Supplementary Figure S4 | Electrochemical performance of a Na-half cell assembled using the Sn metal used as a precursor. (a) Galvanostatic discharge–charge curves measured at 100 mA g^{-1} and (b) cycle performance.

Supplementary Table S1 | Electrochemical performance of various phosphide-based compounds for SIB anodes.

Material	Synthesis method	Capacity		Cyclability			Rate capability		Ref.
		Voltage (V *vs.* Na/Na^+)	Capacity at 2nd cycle ($mAh\ g^{-1}$)	Current density ($mA\ g^{-1}$)	Cycle number (–)	Retention (%)	Current density ($A\ g^{-1}$)	Capacity ($mAh\ g^{-1}$)	
P@C	Ball milling	0–2.0	~350	200	200	86	0.15	~320	2
FeP	Ball milling	0–1.5	~320	50	60	69	0.5	~50	3
CuP_2/C	Ball milling	0.01–2.5	~450	150	30	96	2	~160	4
CoP	Ball milling	0–1.5	~450	100	25	70	2	~80	5
SnP_3/C	Ball milling	0–2.0	~810	150	150	97	2.5	~390	6
Sn_4P_3	Ball milling	0–1.5	~470	100	100	92	10	~50	7
Sn_4P_3	Solvothermal	0.001–1.5	510	100	100	83	5	420	This work

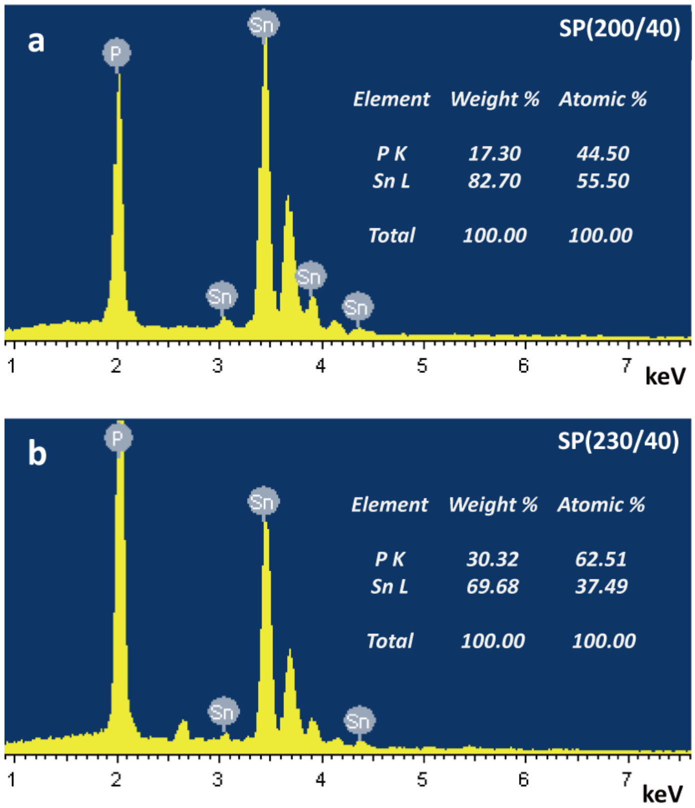

Supplementary Figure S5 | Composition analysis of the solvothermally synthesised Sn-P compound. EDS spectra measured from (a) SP(200/40) and (b) SP(230/40).

References

1. Liang, L. *et al.* Large-scale highly ordered Sb nanorod array anodes with high capacity and rate capability for sodium-ion batteries. *Energy. Environ. Sci.* **8**, 2954-2962 (2015).

2. Wu, N., Yao, H.-R., Yin, Y.-X. & Guo, Y.-G. Improving the electrochemical properties of the red P anode in Na-ion batteries via the space confinement of carbon nanopores. *J. Mater. Chem.* A. **3**, 24221-24225 (2015).

3. Li, W.-J., Chou, S.-L., Wang, J.-Z., Liu, H.-K. & Dou, S.-X. A new, cheap, and productive FeP anode material for sodium-ion batteries. *Chem. Commun.* **51**, 3682-3685 (2015).

4. Zhao, F. *et al.* Nanostructured CuP_2/C composites as high-performance anode materials for sodium ion batteries. *J. Mater. Chem. A.* **3**, 21754-21759 (2015).

5. Li, W.-J., Yang, Q.-R., Chou, S.-L., Wang, J.-Z. & Liu, H.-K. Cobalt phosphide as a new material for sodium storage. *J. Power Sources.* **294**, 627-632 (2015).

6. Fan, X. *et al.* Superior stable self-healing SnP_3 anode for sodium-ion batteries. *Adv. Energy Mater*. **5**, 1500174 (2015).

7. Li, W. *et al.* $Sn_{4+x}P_3$@amorphous Sn-P composites as anodes for sodium-ion batteries with low cost, high capacity, long life, and superior rate capability. *Adv. Mater.* **26**, 4037-4042 (2014).
